# Influence of Rule Manipulation on Technical–Tactical Actions in Young Basketball Players: A Scoping Review

**DOI:** 10.3390/children10020323

**Published:** 2023-02-08

**Authors:** Ricardo André Birrento Aguiar, José Maria Giménez Egido, José Manuel Palao Andrés, Enrique Ortega-Toro

**Affiliations:** 1 Faculty of Sports Sciences, University of Murcia, 30720 Santiago de la Ribera, Spain; 2 Human Movement and Sports Science (HUMSE), Faculty of Sports Sciences, University of Murcia, 30720 Santiago de la Ribera, Spain; 3 Sports Performance Analysis Association (SPAA), Faculty of Sports Sciences, University of Murcia, 30720 Santiago de la Ribera, Spain; 4 Faculty of Education, University of Alicante, 03690 Sant Vicent del Raspeig, Spain; 5 Health, Exercise Science and Sport Management, University of Wisconsin-Parkside, Kenosha, WI 53144, USA

**Keywords:** sport, children, training, competition, scaling equipment

## Abstract

The purpose of this scoping review was to analyse the effect of rules modification on technical and tactical action in young basketball. The publications search period ranged from January 2007 to December 2021. The search covered the following electronic databases: SCOPUS, SportDiscus, and the Web of Science core collection. Following this search process, 18 articles were included in the review. The following variables were analysed: characteristics of the sample, the constraints manipulated, the duration of the intervention, and the effect on technical–tactical actions. The studies reviewed modified the following constraints: (a) number of players (66.7%), (b) court dimensions (27.8%), (c) ball/player interactions (11.1%), and (d) ball/player interactions, basket height, game time and number of baskets (5.6%, respectively). The findings show that rule manipulation can increase players’ participation and promote the variability of players’ actions. The current evidence about rule modification in youth basketball presents areas in which more studies are needed to have a complete perspective of their impact in practice and competition through the different stages of players’ development. Taking into account individual needs and developmental stages, further studies should consider different age groups (e.g., from U-10 to zU-14) and female players. Expanding scientific knowledge in this area would help coaches make short- and long-term plans in accordance with players’ developmental stages.

## 1. Introduction

Sports rules establish how the game is played. It is common for coaches and federations to realize adaptations of the official rules to get different goals, such as facilitating learning, promoting different actions, scaling the game to children, or developing specifical physical capacities (e.g., the inclusion of the three-point line in basketball). However, although these adaptations are common, most of the research done about these adaptations has been done in adult sports [1,2,3,4,5]. There is reduced evidence of the impact of rule manipulations on children and the developmental stages of the players. This involves the absence of strong evidence about the impact of manipulating the task and the practice environment constraints in youth sports. The present review attempted to provide information about the current state of rule modification in basketball and provide recommendations about possible research lines to follow.

Sports rules establish the task and environment constraints (e.g., the number of players or court size). A manipulation of constraints that establish the official rules has the goal of creating more optimal learning landscapes and enhancing players’ teaching-learning processes [6,7,8,9,10,11,12]. Rule manipulation is done by coaches and sports stakeholders to adapt practice and competition to players’ characteristics. This manipulation can help to facilitate the holistic development of players in competition and training [13,14,15,16,17,18]. For example, the use of small-sided games in which there is a manipulation of the court size, and the number of players is common in practice. This approach is an effective strategy for training technical and tactical skills in different ages and skill levels [19,20,21,22]. These manipulations affect the participation and actions of players and teams (e.g., increase of the 1 vs. 1 situations). With their use, coaches are promoting the realization of different actions and behaviours by players in training. However, the impact depends on the type of modification, the age of players, and their skills (among others).

Most of the current research is focused on adults and the study of the impact of rule manipulation on physical actions (e.g., external workload). There is less evidence on the impact of common manipulations of the task and environment constraints implemented by coaches and other stakeholders, such as the number of players, court size, basket height, dribbling rules, or type of defence. These limitations show that is not clear how the rule changes in practice and competition affect technical and tactical actions, their efficacy, and the decision-making of basketball players of different ages and levels [4,15,23,24,25,26,27,28,29,30]. Besides, each study shows the effect of different rule manipulations on players of specific ages and levels. It is necessary to have a broad perspective of the effect of these rule modifications in practice and competition through the different stages of player development to provide evidence-based information that can be referred to in the process of their implementation. This information can also help sports scientists to establish future research according to the deficits found in the literature. The purpose of this scoping review was to analyse the effect of rule modification on technical and tactical action in young basketball players.

## 2. Materials and Methods

This scoping review was conducted in accordance with the Preferred Reporting Items for Systematic Reviews and Meta-Analyses (PRISMA) Statement [31,32].

### 2.1. Search Strategy

The search conducted for this study covered the following electronic databases: SCOPUS, SportDiscus, and the Web of Science core collection (e.g., the Social Citation Index Expanded, the Social Sciences Citation Index, and the Emerging Sources Citation Index). The following keyword combinations were used: TS = (BASKET*) AND TS = “Small-sided games” OR “large-sided games” OR “large-sided games” OR “Short small-sided games” OR “small-sided basketball games” OR “small-sided and conditioned games” OR “Equipment scaling” OR “Scaling sporting equipment” OR “Scaling basketball equipment” OR “Scaling constraints” OR “modified basketball games” OR “Modifying equipment” OR “Modified games” OR “Changing rules” OR “ Reducing pitch size”.

### 2.2. Article Screening and Data Extract Process

**Figure 1:**
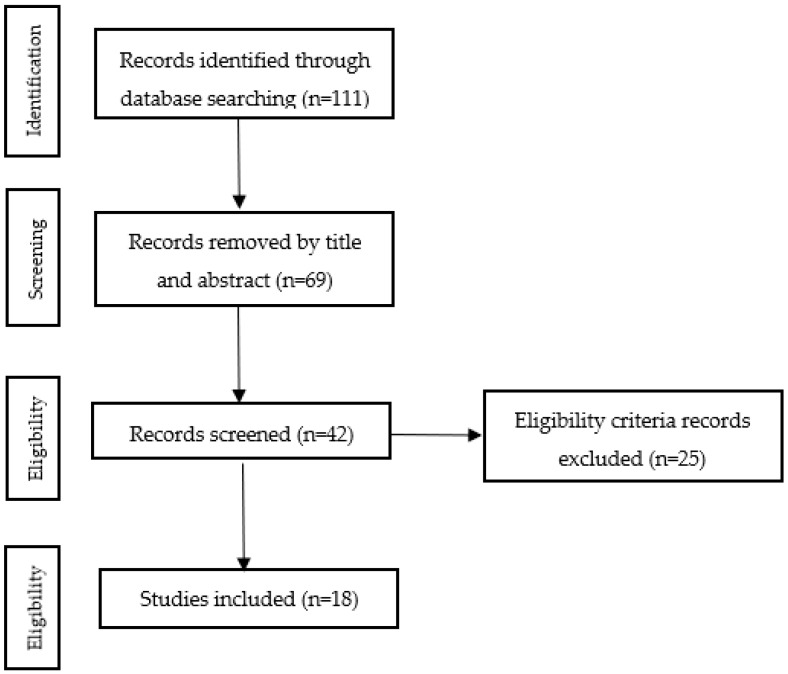
The PRISMA flowchart illustrating each stage of the literature search.

The publication search period ranged from January 2007 to December 2021. In total, 111 papers were identified in the original database search. Two independent reviewers selected the abstracts and full texts of the studies that met the inclusion criteria. With regards to papers where there was doubt on whether they met the inclusion criteria, a third expert reviewer made the decision instead. Reliability was calculated in the registry using Cohen’s Kappa coefficient, whereby minimum values of 0.99 were obtained. With regards to the selection and extraction studies process, the following hierarchical order was utilized: excluded by title; excluded by abstract; and excluded by full text that did not meet the eligibility criteria. Figure 1 details the data extraction process.

### 2.3. Eligibility Criteria

The following eligibility criteria were used, where the papers included: (a) small-sided games in basketball; (b) modified and changing rules; and (c) was written in English, Portuguese, or Spanish. The exclusion criteria utilized were studies that included: (a) other sports as the subject of study; (b) wheelchair basketball; (c) adult players; (d) did not analyse technical–tactical variables; (e) congress papers; (f) systematic reviews; (g) nonexperimental studies; and (h) a sample of coaches and/or teachers.

### 2.4. Variables Analysed

The following variables for each paper were analysed: (a) publication year; (b) sex (women, men, and both sexes participated); (c) age of the sample (under 10, under 12, under 14, under 16, and under 18); (d) duration of the study (short-term: less than four weeks, medium-term: between 1 and 4 months, and long-term: more than 4 months); (e) constraints modification (basket height, court dimensions, game time, number of baskets, number of players, ball/player interactions), (f) technical–tactical variables studied (dribbles, passes, shots, rebounds, ball screens, decision-making, steals, turnovers, offensive possessions, fakes, assists, fast breaks, and defensive actions), and (g) effect of constraint manipulation on the technical and tactical variables.

## 3. Results

### 3.1. Studies Characteristics

Only a total of 18 articles matched the inclusion criteria. Most of the studies were done in the last years of the period analysed (61.1% in the last four years) and most were done by researchers from Portugal, Brazil, and Italy. Most of the studies included older categories (72.2% for U-16 and U-18). Twenty-two per cent of the studies included female players, 53% male players, and 25% involved players of both sexes. Regarding the duration of the study, no studies were found with an intervention duration of longer than four months. Fifty-six per cent had a short duration (less than four weeks) and 44% had a duration between one month and four months.

The studies reviewed modified the following constraints: (a) number of players (66.7%), (b) court dimensions (27.8%), (c) ball/player interactions (11.1%), and (d) ball player interactions, basket height, game time and number of baskets (5.6%, respectively). These studies analysed the effect of manipulating these constraints on 15 technical–tactical variables. Three were used in more than 50% of the studies, which were: pass and shot (58.8%) and rebound (52.3%). Another group of variables was used in more than 20% of the studies, which were: dribble and offensive possessions (35.3%), turnovers (29.4%), and steals (23.5%). The least studied variables with a presence of less than 20% in the studies were: defensive actions (17.6%), assists (11.8%), as well as ball screens, decision-making, fakes, and fast-break (5.8%). Specific information on the results (authors, sample, independent variables, dependent variables, and the effect of the intervention for each study) can be found in Appendix A (Table A1). 

### 3.2. Constraints Modification: Number of Players

Four studies analysed the differences between playing 3 vs. 3 and 5 vs. 5 [33,34,35,36]. The games with fewer players showed more situations with potential for learning and improving decision-making and motor skills, due to the higher number of 2-point shots, possessions, and passes. Two studies compared the difference between playing 2 vs. 2 and 4 vs. 4 [21,37]. The 2 vs. 2 situation showed a higher number of ball screens, dribbles, passes, rebounds, and shots. In addition, in the study done with younger players, there were a higher number of dribbles, rebounds, passes, and shots [21]. A similar study compared the 3 vs. 3, 4 vs. 4, and 5 vs. 5 situations [22]. They found a higher number of assists, passes, rebounds, steals, shots, and turnovers in 3 vs. 3 games than in the 4 vs. 4 and 5 vs. 5 games.

Two studies compared the different options of player number manipulation: 1 vs. 1, 2 vs. 2, 3 vs. 3, 4 vs. 4, and 5 vs. 5. These studies also manipulated the court dimensions in each situation [38,39]. In male players and female players [38,39] situations with fewer players (and court size) had higher numbers of technical–tactical actions per player per minute. However, both studies found that each situation involved an increase of different actions (e.g., a higher number of rebounds on 2 vs. 2, a higher number of shots on 4 vs. 4, or a higher number of turnovers on 1 vs. 1).

One study focused on the analysis of how players improved playing 2 vs. 2 after six weeks of training [40]. The results showed a higher number of shots and more passing efficacy at the end than on the first day of the intervention. Two studies focused on the effect of having more players on one team [23,41]. One study compared the situations 3 vs. 2, 3 vs. 3, and 3 vs. 2+1 [41]. Its results showed that playing 3 vs. 2 involved more effective dribbles and rebounds when compared with 3 vs. 3 and 3 vs. 2+1. Another study analysed the differences between the situations 3 vs. 3, 4 vs. 3, and 3 vs. 3+1 (non-scorer floater) [23]. Its results showed a higher number of dribbles, passes, fast-breaks, offence possessions, and space creations without the ball, compared to the 4 vs. 3 and 3 vs. 3+1 situations.

### 3.3. Constraints Modification: Court Dimensions

Five studies modified the court dimensions [37,38,42,43,44]. In four of these, researchers compared the difference between playing in half and full courts [37,42,43,44]. In the other study, researchers manipulated the court dimensions simultaneously with the number of players per team (15 × 6 m, 22 × 8 m, 24 × 11 m, 26.13 m, and 28.15 m) [38]. These studies showed an increase in the number of actions of dribble, pass, rebound, shot, and turnover, and an increase in the number of fakes.

### 3.4. Constraints Modification: Ball/Player Interactions, Basket Height, Game Time and Number of Baskets 

Regarding the manipulation of the basket, two studies focused on this aspect. One study focused on the effect of basket height and the other study on the number of available baskets. The study that focused on basket height assessed the differences between playing 5 vs. 5 with a basket of a height of 3.05 m and a height of 2.80 m [45]. The study showed that with a lower basket height, there were a higher number of fast breaks and longer positional attack phases. The researchers found an increase in the number of defensive and offensive rebounds, dribbles, and shots in the four baskets games. However, there was a decrease in the number of passes, number of steals, and number of possessions in the four baskets games.

One study focused on the effect of game time (4 × 2.5 min and 2 × 5 min) on individual and team offence and defence actions [37]. The study found that playing for shorter periods increased the realization of dribbles, passes, shots, rebounds, and ball screens. Regarding the manipulation of the interaction of the players with other players and the interaction with the ball, two studies were found. One focused on defensive pressure [46]. This study found that changing the type of defence pressure during the game promoted different collective offence behaviours, which affected the creative and perception actions done by players. The second study assessed the difference between playing with dribbling and without dribbling [22]. In this study, the results showed an increase in the number of assists, passes, rebounds, steals, and turnovers in the no-dribbling games, and a decrease in the number of shots in no-dribbling games.

## 4. Discussion

### 4.1. Studies Characteristics

The low number of studies found with younger athletes demonstrates the need for increasing our knowledge about the effect of rule manipulation in the foundational stages of basketball players’ development. Most of the studies in the review used a sample of under-16 and under-18 players. There is a paucity of studies at the under-10, under-12, and under-14 levels. Similar issues occur with the study of rule modification in young female basketball players. We should obtain information on all the different stages of the developmental process to guide the training and competition decisions (e.g., what rules we can change to promote players’ learning). The analysis of this sample of studies showed important differences in the ways that different researchers report the characteristics of the players. It is recommended that standard criteria regarding their maturation, skills, level of competition, and hours of practice are included in future studies to allow other researchers to contextualize the studies. An adaptation to youth athletes as proposed by Alannah et al. could be a starting point [47]. We know that practice and competition at young levels should be in line with the evolutionary process of each player at each stage of development [2,35,48]. However, it is not possible to achieve this if we do not have information on the maturational development, skills, experiences, etc. of the players [49,50,51]. Regarding the duration of the studies, there is a paucity of longitudinal studies. This limits our knowledge about the effectiveness of the changes in the long term. If it is not possible to implement longitudinal studies, another possibility is to study the effect of the changes in different age groups, sexes, or skill levels at the transversal level.

### 4.2. Constraints modification: Number of Players

A reduction in the number of players involves an increase in the actions done by players [21,33,34,35,36,37]. These results can be considered normal; fewer players per team involves more ball contact by players (dribble, rounds, passes, and shots). If the practice infrastructure allows it (e.g., multiple baskets available), this manipulation can increase the players’ participation in offence and defence. However, if several baskets are not available, this approach may involve not all players participating. The manipulation of player numbers can be done simultaneously with the court size reduction. Unfortunately, this aspect has been studied in only two studies [38,39]. They found that 1 vs. 1 and 2 vs. 2 situations increased turnovers and rebounds. The reduction of players and space could cause players to assume more risk in their actions to solve the game situations. This issue was not found for the 3 vs. 3 situations. In summary, our current evidence shows that the manipulation of the number of players can involve an increase in player participation in training and competition.

Another possibility evaluated in several studies was the effect of having different numbers of players in a team or having a non-scorer floater player [23,41]. This type of manipulation means that the team with more players has a higher number and more effective offensive actions, as well as defensive actions. This manipulation also involves the realization of more fake situations by players. These results can be considered normal. The creation of an unbalance situation for one team facilitates the realization of their individual and team actions, as well as introduces more variability. This allows players to have a higher number of positive actions, which can increase their acquisition. For players in the team with fewer players, this type of approach is a challenge to overcome. Although the number of studies in this research was limited [23,41], our current evidence shows that these manipulations can be used by coaches to promote positive and variable individual and team actions, as well as challenge players in defence.

### 4.3. Constraints Modification: Court Dimensions

A reduction of the court dimensions involves an increase in individual and team actions [37,38,42,43,44]. The consequence of this could be that with more space available, players do more collective actions, which provokes a higher number of individual skills, which results in the higher participation of players. The reduction of space involves the realization of more fake movements by players in order to overcome opponents. As we mentioned in the previous point, more studies are needed to determine the relationship of court dimension manipulation with other variables, such as the number of players, the type of defence, or no-dribbling. The implementation of the court dimension changes is easier when the standard court dimensions are used (half-court or full-court), so most of the studies focused on this approach [37,42,43,44]. Only one study focused on other sizes and combined them with manipulation of the number of players. Smaller court dimensions (and numbers of players) involved more shots and rebounds [38]. The reduction of court dimensions could provoke a high number of 1 vs. 1 situations, which involve the possibility of greater variability of movements [45]. Our current evidence shows that more studies are needed to increase our knowledge about different possibilities that involve the manipulation of court dimensions according to the number of players, and the creation of different zones or the alteration of the current ones (e.g., three-point line and paint-zone).

### 4.4. Constraints Modification: Ball/Player Interactions, Basket Height, Game Time and Number of Baskets

There was a reduced number of studies that manipulated baskets available, game time, and players’ actions in defence and with the ball [22,37,46,52]. All these studies showed an increase in individual and team offence skill actions. Manipulations, like playing for numerous short periods of time, using the pressure defence, or not allowing dribbling, are interesting tools that coaches can use in practice to generate variability, challenge decision-making and promote different behaviours in players (e.g., the realization of more passes when no dribbling is allowed). However, more studies are needed to confirm these findings because only one study was found for each of these manipulations.

Only one study out of 18 focused on the manipulation of basket height. The rest of the studies used the same basket height used for adults, independently of the characteristics and age of the youth players studied. Most of the studies focused on the manipulation of actions that were easy to implement or transfer to practice and competition. Manipulations that involved changing the court lines or manipulating the basket height were fewer. However, the study that evaluated the effect of playing with a lower basket (2.80 m) found changes in the offensive game style. There were more fast breaks and longer positional attacks, which involve higher numbers of 1 vs. 1 situations and more player participation. It is critical to increase our evidence about which basket height is more appropriate for each age group, sex, and skill. At this level, no studies were found regarding the manipulation of ball size. This information about equipment scaling could help to establish if it should be introduced in competitions and the benefits of using different basket heights (or ball sizes) in practice.

### 4.5. Future Research and Practical Applications

The analysis of the studies reviewed shows that there are areas in which more research is needed, such as for U10, U12, and U14 age groups, female basketball, and long-term studies (more than four months). Our current evidence does not allow us to have a complete perspective of the effect of different constraining manipulations throughout the whole process of basketball players’ development. At this level, it is important that researchers better contextualize the characteristics of their sample (e.g., skills/level, hours of practice). Regarding the different constraints that coaches can manipulate, most of the studies focused on the manipulation of the number of players (e.g., 3 vs. 3) and official court dimensions (full-court versus half-court). More research is needed regarding other less common manipulations. For example, how the basket height should be adapted through the change of player height during their maturation. Another aspect to consider in future studies is that most of the studies analysed individual actions done by players. It is recommended that we gain a better perspective of the impact of the changes, and the impact on collective defence and offence actions (e.g., fast-break, offence possession, spacing with and without the ball, type of defence, defence team movements).

From a practical perspective, the studies showed how each manipulation involves specific changes in technical and tactical actions. This information can provide coaches with a better understanding of the impact of reducing the number of players or playing half-court versus full-court. It is necessary to emphasize that there is a reduced number of studies in some age groups and female basketball, and the information about the sample used in some studies limits the generalization of the findings. However, the trends that these studies provide can serve as a reference to guide coaches in the design of task and practice situations that can increase players learning. The reduced evidence-based information about youth basketball limits the analysis and discussion of whether the current competition rules used in developmental stages need to be reconsidered or not.

## 5. Conclusions

The current evidence about rule modification in youth basketball presents areas in which more studies are needed to have a complete perspective of their impact in practice and competition through the different stages of players’ development. Taking into account individual needs and developmental stages, further studies should consider different age groups (e.g., from U-10 to U-14) and female players. Expanding scientific knowledge in this area would help coaches make short- and long-term plans in accordance with players’ developmental stages. It is also necessary to include in the studies that analyse technical–tactical actions, variables related to defence actions, team actions, and decision-making. The findings show that rule manipulation can increase players’ participation and promote the variability of players’ actions. The authors of this study believe that the scientific community, sports federations, and sports clubs should promote and conduct more studies in this line of research for all age groups and sex.

## Data Availability

No new data were created or analyzed in this study. Data sharing is not applicable to this article.

## Appendix A

**Table A1:** Article analysed.

Citation	Sample	Duration Intervention	Independent Variable	Technical Tactical Variables	Mean Findings
Leite, Leser, Gonçalves, Baca & Sampaio (2014) [46]	20 male basketball players (under 16.05 ± 2.09 years)	Short-term (1 day)	Type of defence pressure on 5 vs. 5	Defence and offence possessions.	Changing the level of defensive pressure promotes different collective behaviours.
Tallir, Pihilippaert, Valckle, Musch & Lenoir (2012) [36]	23 male and 7 female basketball players (11.08 ± 0.55 years old).	Short-term (2 days)	3 vs. 3 and 5 vs. 5	Decision-making.	3 vs. 3 involved more potential situations for learning (and improving) decision-making and motor skill execution.
Mateus, Gonçalves, Exel, Esteves & Sampaio (2019) [52]	14 male basketball players (14 ± 0.9 years old)	Short-term (2 days)	2 and 4 baskets	Dribble, passes, offence positions, rebounds, & steals.	Short-term effects found in changing the number of baskets provoke offence technical variables (dribble, pass, and rebound), defence technical variables (rebound and steal), and team offence variables (offence possessions).
Ortega, Garcia-Angulo, Gimenez & Palao (2021) [45]	51 male basketball players (13.32 ± 0.41 years old).	Short-term (2 days)	Basket Height: 3.05 m vs. 2.80 m	Fast breaks, passes, possessions, & shots.	The use of an adapted basket height in the competition promoted a game style that increased the occurrence of fast breaks and the execution of long positional attack phases.
Bredt, Camago, Mortoza, Andrade, Paopuci, Rosso, Praça & Chagas (2021) [23]	45 male basketball players (14.2 ± 0.3 years old).	Short-term (3 days)	3 vs. 3, 4 vs. 3 and 3 vs. 3+1	Dribbles, fast breaks, offence possessions (no shots), space creations wo ball, possessions, & passes.	The small-sided games with additional players or those games played on a half-court enhance group tactical–technical behaviour.
Sanchez, Carretero, Valiente, Gonzalo, Sampaio & Casamichana (2018) [44]	6 young female basketball players (14.3 ± 0.5 years old).	Short-term (4 days)	Half-court and-full court	Passes, possessions, rebounds, & shots.	Half court games involved a higher number of passes, possessions, rebounds, shots
Alti, Koklu, Alemdaroglu & Kocak (2013) [42]	12 young female basketball players (age 15.5 ± 0.5 years old).	Short-term (1 week)	Half-court and full-court	Assists, passes, rebounds, shots, steals, passes, & turnovers.	Half-court 3-a-side games involved a higher number of technical actions compared to full-court 3-a-side games.
Erculj, Vidic & Leskosek (2020) [33]	72 teams (≈500 male and female U18 basketball players)	Short-term (1 week)	3 vs. 3 and 5 vs. 5.	Shots.	3 vs. 3 basketball created more shooting situations and provoke a higher number of shots.
López & Arias (2019) [34]	18 mini-basket players (9.89 ± 0.8 years old).	Short-term (4 weeks)	3 vs. 3 and 5 vs.5.	Offence possessions & passes.	The 3 vs. 3 game form should be used to favour players’ game implications (passes and offence possessions), successful participation, positive emotions, as well as satisfying preferences.
Diniz, Bredt & Praça (2021) [41]	45 schoolchildren (11.55 ± 0.49 years old) from both sexes.	Short-term (4 weeks)	3 vs. 3, 3 vs.2 and 3 vs. 3+1.	Dribbles, passes, rebounds, & shots.	3 vs. 2 small-sided games involved higher participation than 3 vs. 3 and 3 vs. 3+1 small-sided games. The constant presence of a free player in these types of small-sided games may variability for shooting and rebounding.
McCormick, Hannon, Newton, Shultz, Miller & Young (2012) [35]	12 young male basketball players (15 years old).	Medium-term (6 weeks)	3 vs. 3 and 5 vs. 5	Possessions.	The results suggest that 3 vs. 3 leagues may be an appropriate sport for initial exposure to young basketball players.
Klusemann, Pyne, Foster & Drinkwater (2012) [37]	16 elite junior basketball players. 8 male (18.2 ± 0.3 years old) and 8 female (17.4 ± 0.7 years old).	Medium-term (6 weeks)	2 vs.2 and 4 vs.4. Half court and full court 4 × 2.5 min vs. 2 × 5 min	Ball screens, dribbles, passes, shots, rebounds, and ball screens.	The number of players on the court, court size, and work-to-rest ratios involved changes in the realization of technical actions and their Frequency.
Delextrat & Martinez (2014) [40]	24 male basketball players (U17)	Medium-term (6 weeks)	2 vs. 2	Defensive actions, pass, & shot.	The use of 2 vs. 2 increased basketball-specific skills performance.
Conte, Favero, Nierderhausen, Capranica & Tessitore (2016) [21]	21 young male basketball players (15.4 ± 0.9 years).	Medium-term (6 weeks)	2 vs. 2 and 4 vs.4.	Dribbles, passes, rebounds, shots, steals & turnovers.	The number of players influenced the technical actions done in the drills. The 2 vs. 2 situation increased ball screens, dribbles, passes, rebounds, and shots.
Clemente, Conte, Sanches, Moleiro, Gomez & Lima (2018) [39]	20 young male basketball players (U12 & U14)	Medium-term (5 weeks)	1 vs. 1, 2 vs. 2, 3 vs. 3, 4 vs. 4 and 5 vs. 5	Rebounds, shots, & turnovers.	The number of players influence the physical effort of players which was associated with tehcnical actions (received & attacking balls and shots).
Bredt, Torres, Diniz, Praça, Andrade, Morales, Rosso & Chagas (2020) [43]	12 junior male basketball players (17.01 ± 0.24 years old).	Medium-term (5 weeks)	Half court and full court.	Defensive actions, dribbles, & fakes.	3 vs. 3 full court increased the number of fakes.
Ferioli, Rucco, Rampinini, La Torre, Mnafredi & Conte (2020) [22]	10 male basketball players (average 18.3 years old).	Medium-term (16 weeks)	3 vs. 3, 4 vs. 4 and 5 vs. 5 Regular dribble and No dribble.	Assists, passes, rebounds, shots, steals & turn-overs.	Reducing the number of players increased the game-based drills and technical elements, while the No Dribble format promoted a greater number of turnovers and passes.
Clemente, Bredt, Praça, Andrade, Sanchez, Moleiro & Lima (2021) [38]	10 female basketball players (14.3 ± 1.3 years old).	Medium-term (15 weeks)	1 vs. 1, 2 vs. 2, 3 vs. 3, 4 vs. 4 and 5 vs. 5. 15 × 6 m, 22 × 8 m, 24 × 11 m, 26 × 13 m and 28 × 15 m (respectively).	Rebounds & shots.	The smaller basketball small-sided games format induced higher numbers of technical–tactical actions per player per minute. Furthermore, they induced adjustments in the relative playing area. Regarding the effect of successive small-sided games bouts, two successive bouts only do not seem to impact either the numbers of technical–tactical actions or RPE.
